# Breast Cancer Survival in Eastern Region of Ghana

**DOI:** 10.3389/fpubh.2022.880789

**Published:** 2022-06-02

**Authors:** Paddy Ssentongo, John S. Oh, Forster Amponsah-Manu, William Wong, Xavier Candela, Yubraj Acharya, Anna E. Ssentongo, Daleela G. Dodge

**Affiliations:** ^1^Department of Public Health Sciences, Penn State College of Medicine, Hershey, PA, United States; ^2^Department of Surgery, Penn State Hershey Medical Center, Hershey, PA, United States; ^3^Department of Surgery, Eastern Regional Hospital, Koforidua, Ghana; ^4^Department of Health Policy and Administration, College of Health and Human Development, The Pennsylvania State University, University Park, PA, United States

**Keywords:** breast cancer, survival, Africa, Ghana, epidemiology

## Abstract

**Objective:**

Five-year overall survival rate of breast cancer in low-income countries (LICs) is significantly lower than in high-resource countries. This study explored clinical and pathological factors influencing mortality in the Eastern region of Ghana.

**Methods:**

We performed a retrospective medical chart review for patients undergoing surgery and chemotherapy for breast cancer at a regional hospital in Ghana from January 2014 to January 2017. Descriptive and survival analysis was done.

**Results:**

One hundred and twenty-nine patients were included in the study. The median age at presentation was 51 years. Sixty percent of patients presented with poorly differential histological grade III. The most common histological type was invasive ductal carcinoma (83%). Based on stage assessment using only tumor size and lymph node status, 60% presented at stage 3. Only 25% were tested for hormone receptor proteins and HER2 status. Of these, 57% had triple-negative breast cancer (TNBC). The 3-year overall survival rate was only 52%.

**Conclusion:**

The cumulative 3-year survival was 52%. Despite success in reducing cancer mortality in northern Africa, survival in sub-Saharan Africa remains poor. A significantly higher percentage of GIII and TNBC is found in breast cancers seen in Ghana. When combined with limited capacity for accurate diagnosis, cancer subtype analysis, adequate therapy, and follow-up, late-stage presentation leads to poor outcomes. Future studies should emphasize the identification of barriers to care and opportunities for cost-effective and sustainable improvements in diagnosing and treating breast cancer in LICs.

## Introduction

Breast cancer (BC) mortality rates are significantly higher in low-and-middle-income countries (LMICs), such as those in sub-Saharan Africa, than in high-income countries (HICs) (1, 2). Breast cancer is well-studied in HICs and issues of access to healthcare in rural or underserved areas have been studied in this demographic. However, the epidemiology of BC is not well-studied in low and middle-income countries (LMICs), especially in the underserved/rural areas (3). Delayed clinical presentation, limited access to treatment, geographical location (4), stage of the disease (5), tumor biology (6), and access to care (7) all contribute to the high mortality rates in LMIC (8). Breast cancer outcomes, especially mortality rates, need to be studied to define the scope of the issue, which will establish the need for protocols for exploring risk factors and opportunities for intervention.

Eastern Ghana represents an underserved region of sub-Saharan Africa where the treatment results have not been well-studied. The prevailing hypothesis in such settings is that late presentation accounts for poor long-term outcomes. The main objective of this study was to investigate the 3-year overall survival of breast cancer patients treated at the Eastern Regional Hospital of Ghana. While Koforidua is an urban setting, the Eastern Regional Hospital is a referral hospital which serves the population of the rural eastern region where many patients must travel hours to obtain medical care. We sought to characterize the regional epidemiology of breast cancer by assessing age at onset, tumor grade, and biology.

## Methods

### Sampling of Study Participants

We conducted a retrospective cohort study with a consecutive sampling of all women who presented with breast cancer diagnosis from January 2014 to January 2017. The study was conducted at Eastern Regional Hospital of Koforidua (ERHK), a referral hospital of the eastern region of Ghana, serving ~3 million people. It is the referral hospital of 26 district hospitals and has a 364-bed capacity. All patients received breast cancer surgeries by one surgeon, 80% chemotherapy, 20% anti-hormonal therapy, and about 20% radiotherapy (patients all had to travel to Accra for treatment).

An offsite staff pathologist prospectively analyzed Formalin-fixed and paraffin-embedded blocks for the participants for the histopathological classification. The National Health Insurance Scheme (NHIS) does not cover pathology. The specimens must be transported by the patient or their family to St. Joseph's hospital located two miles away and analysis requires an out-of-pocket cost which often leads to delays and in some cases the specimen is discarded—cases with no available pathology were not included in this analysis. The time of specimen fixation is about 24 h. The average time for the final anatomic pathology report to be released is between 30 and 60 days. Immunohistochemistry analysis incurs an extra charge. When performed, during the study period, the slides were either sent to a pathologist in England with the results taking an average of 9 months before completion. Currently, slides for immunohistochemistry are sent to Kumasi with an average 1-month turnaround. Patients were followed for 1.5–4.5 years, from January 1, 2014, to June 2018. Patients were followed or contacted periodically during the follow-up period, either through outpatient clinic visits, chemotherapy appointments, or phone calls. If patients could not be reached, caretakers were contacted to ascertain death/survival. The index date for the survival calculation was determined to be the first date of the histologically confirmed breast cancer diagnosis. Death was the event outcome. Patients who were alive by the closing date of June 2018 and those lost to follow-up were censored. The study was approved by the Pennsylvania State College of Medicine institutional review board and ethical review board of Mount Crest University and Eastern Regional Hospital of Ghana.

### Treatment Algorithms

All patients underwent breast cancer surgery and ~80% of patients received chemotherapy secondary to locally advanced disease. Patients with resectable disease received mastectomy with axillary dissection. With late-stage presentation, which is common in the LMIC population, mastectomies were performed for palliation, when possible (technically feasible). The Eastern region of Ghana has no combined multidisciplinary clinic to treat breast cancer. The Eastern Regional Hospital in Koforidua, Ghana houses the only chemotherapy treatment facility in the region. Chemotherapy cost is not covered by the NHIS. In Koforidua, the JEAD foundation—a breast cancer charitable foundation that funds community outreach and reimburses treatment expenses established by a local breast cancer survivor—covers the cost of chemotherapy for many of the patients who cannot afford to pay for their treatment. Those on chemotherapy received an average of 6 cycles of combination chemotherapy, which most often include cyclophosphamide, adriamycin and 5-fluorouracil (CAF). Estrogen receptor (ER), progesterone receptor (PR)-positive patients and some of those whose hormone receptor status was not known received tamoxifen or anastrazole anti-estrogen targeted treatment (AET) generally for 5 years. The chemotherapy and AET were prescribed by the surgeon in this cohort. The nearest oncological center is located in Accra more than a 2-h drive away from Eastern Regional Hospital. When radiotherapy was recommended and utilized, patients had to travel to the nearest radiotherapy center, also located in Accra, Ghana's capital city.

### Statistical Analysis

Categorical variables were reported as frequencies and percentages. Continuous variables were reported as medians and interquartile ranges. Survival probabilities were calculated using the nonparametric approach, Kaplan-Meier method of estimating survival. Log-rank test was used to compare survival curves. The Cox regression model was used to estimate survival probabilities while controlling for clinically relevant confounders such as age. The survival analysis was also stratified by the tumor grade. Survival analysis was not stratified by hormone receptor status because data was available in only a small portion of the study group. A 3-year cancer survival rate was defined as the ratio of cancer patients who were still alive (regardless of cancer status) over the total number of patients diagnosed with cancer. All data were analyzed using R statistical software version 3.4.3 (R Foundation for Statistical Computing, Vienna, Austria). A *P*-Value of < 0.05 was considered to denote statistical significance.

## Results

**Figure 1:**
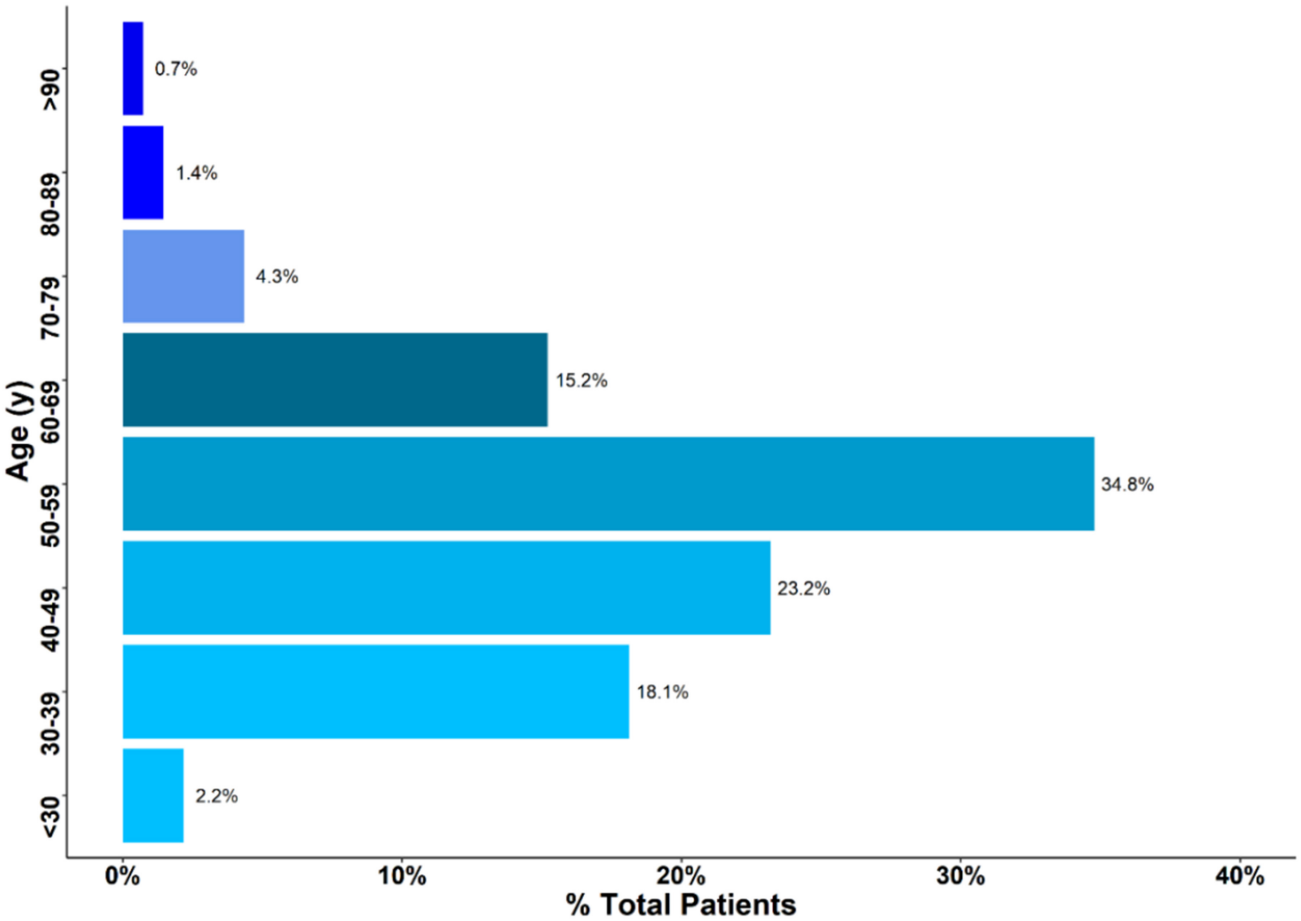
Age of patients presenting with breast cancer. Approximately 76% of Patients were between 30 and 60 years.

This study included a cohort of 129 women diagnosed with breast cancer at Eastern Regional Hospital between 2014 and 2017. The median age at presentation was 51 years (interquartile range 30–75). Approximately 76% of patients were between 30 and 60 years old at the time of diagnosis, and <1% were either younger than 25 or older than 90 (Figure 1).

All the patients had completed at least one previous pregnancy and had one living child. The average number of pregnancies was four, with three living children. Among the patients for whom we could ascertain a breastfeeding history, all except one had breastfed for 1 year or longer. Approximately 32% of patients had a known family history of breast cancer. Regarding co-morbidities, 24% of patients had hypertension and 13% had diabetes mellitus. No patients reported a history of alcohol use or smoking (Table 1).

**Table 1:** Demographics of study participants.

**Variable**	**Number (%)**
Age, median (interquartile range), year	51 (30–65)
Sex (female)	129 (100)
**Grade of breast cancer**	
Grade I	6 (5)
Grade II	44 (34)
Grade III	79 (61)
**Primary breast involved**	
Right	64 (50)
Left	54 (42)
Unknown	11 (8)
Estrogen receptor^*^	16/35 (46)
Progesterone receptor ^*^	5/35 (14)
HER2^*^	5/35 (14)
Smoking^*^	0/38 (0)
Drinking^*^	0/38 (0)
Previous pregnancy^*^	38/38 (100)
Breastfed^*^	38/38 (100)
Family history of breast cancer^*^	12/38 (32)
Type 2 diabetes mellitus^*^	5/38 (13)
Hypertension^*^	9/38 (24)

*HER2, human epidermal growth factor receptor 2*.

* *Data was not available for all patients*.

**Figure 2:**
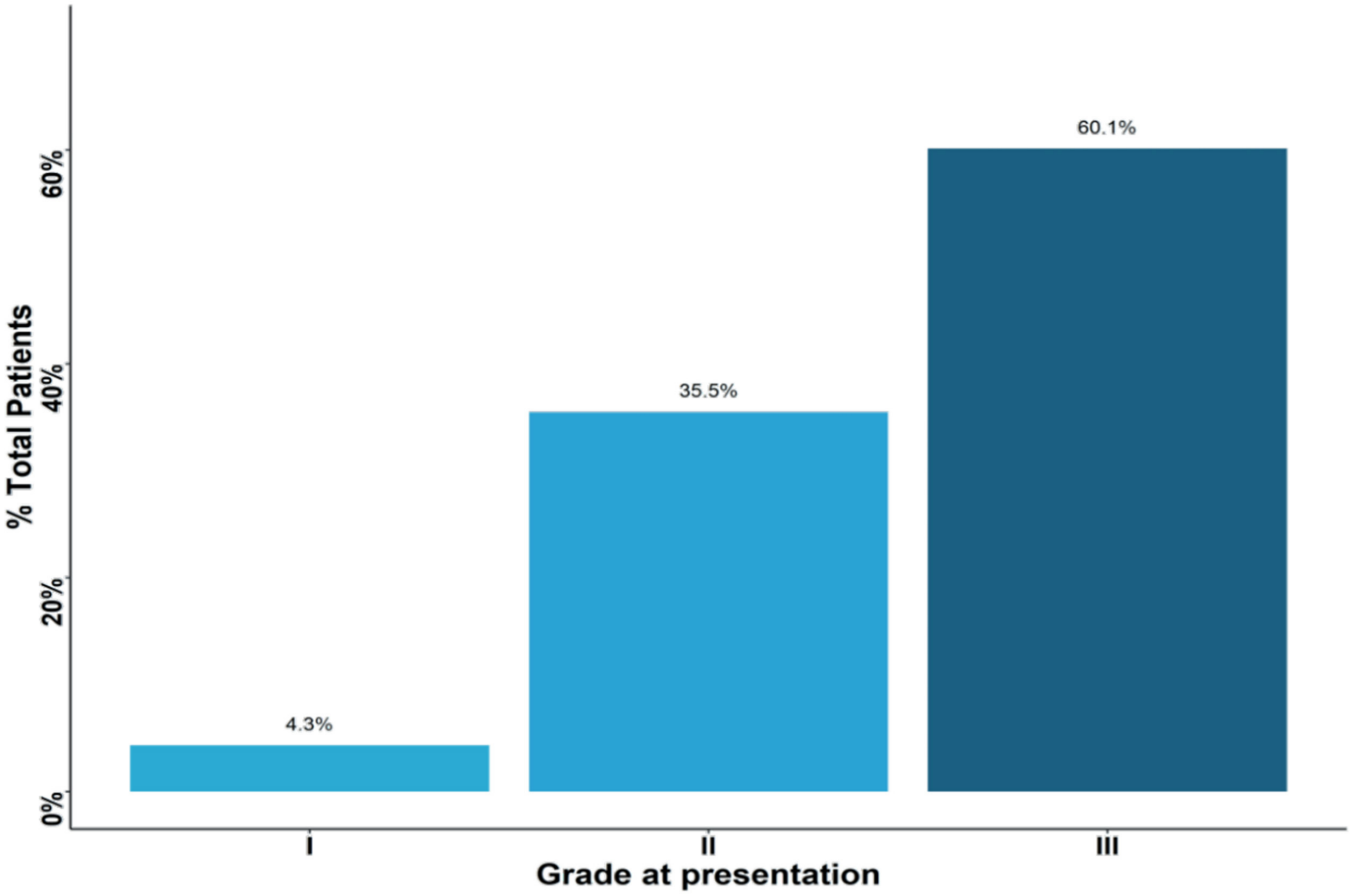
Percent of patients presenting with each grade type. Approximately 60% of patients presented with grade three tumors.

**Figure 3:**
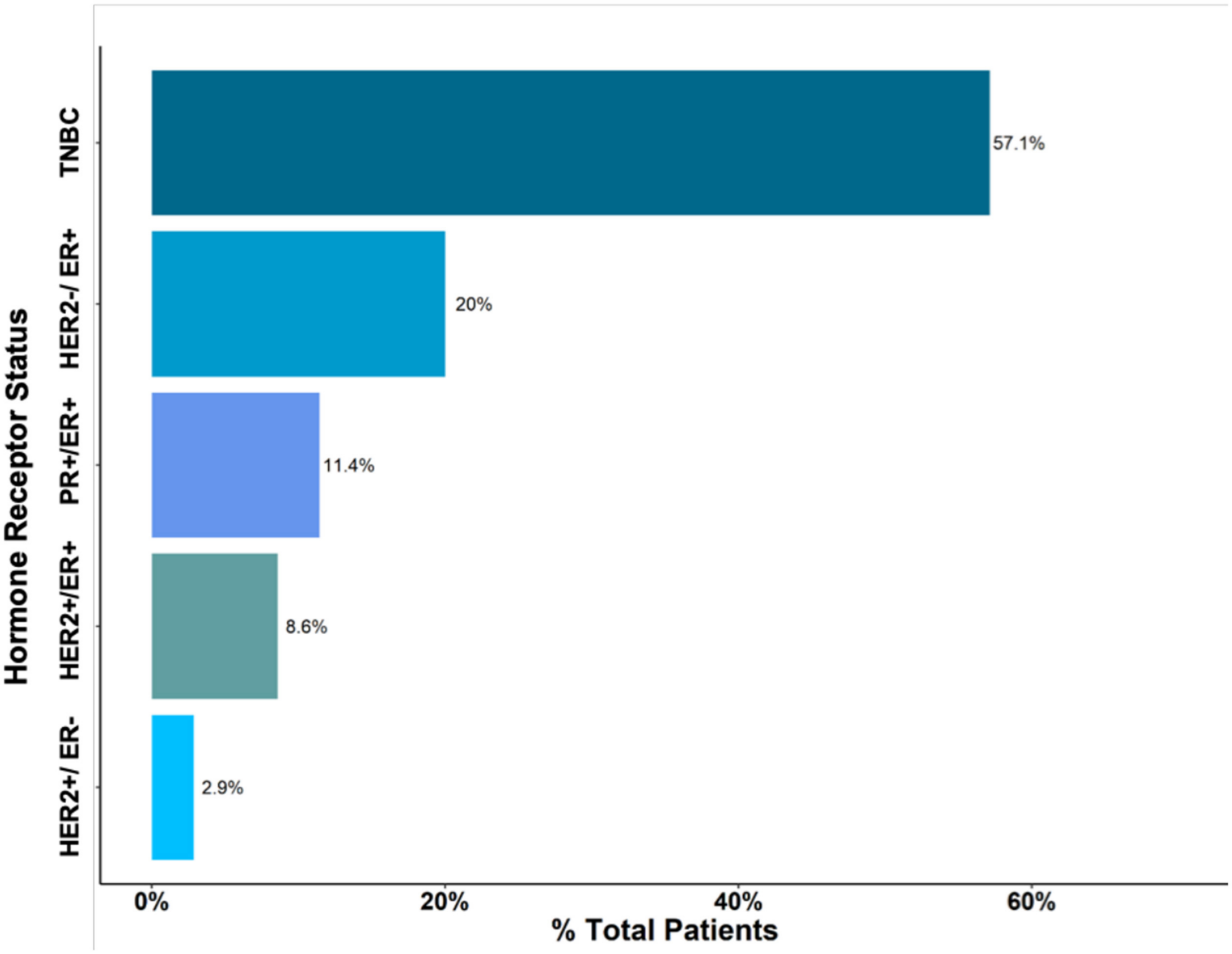
Histological type of Breast Cancer. Triple-Negative Breast Cancer (TNBC) accounted for approximately 57% of cases.

**Figure 4:**
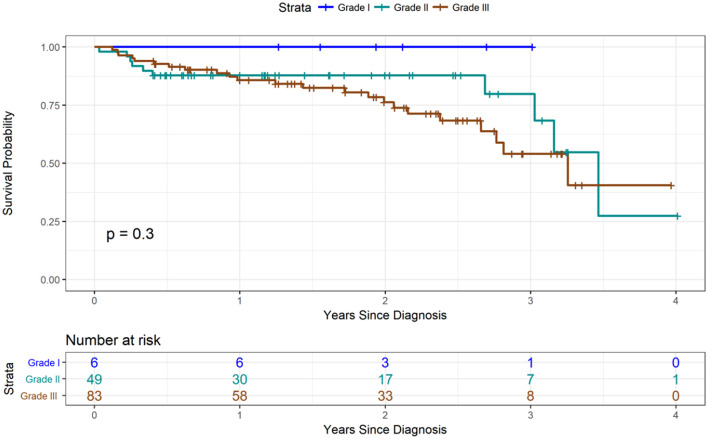
Survival stratified by grade type. Three-year survival was approximately 50%.

Based on histological analysis, <5% of patients had grade I cancer, whereas the rest of patients had either grade II or III breast cancers (Figure 2). The most common histologic type of breast cancer was invasive ductal carcinoma (83%). Only 25% of patients had hormone receptor protein testing on the cancer performed. Among these patients, 57% had a triple-negative breast cancer (Figure 3). All the patients who were tested and found to have hormone receptor-positive tumors were prescribed tamoxifen. However, no data on patient compliance with AET was available. The overall 3-year survival rate was 52% (Figure 4). Although survival was higher in patients with grade I disease compared to grade II and III (HR = 1.5, 95% CI 0.5–2.0, *P* = 0.35), the association of low histologic grade and prognosis did not reach statistical significance. The overall median (IQR) survival was 40 months (range 20–60 months). Forty six percent of the treated patients were lost to follow-up, despite the staff and investigators making significant efforts to reach them or their relatives.

## Discussion

This study is the first to explore breast cancer mortality rates for patients treated in a regional hospital for Eastern region of Ghana. The data primarily highlights three critical issues. First, the survival rates in Eastern Ghana are substantially lower than in HICs and also significantly lower than documented in studies of survival in other regions of Africa (9). Second, patients in this cohort presented more often with advanced grade disease and at a younger age. Finally, the proportion of triple-negative breast cancers (TNBCs) is substantially higher when compared to breast cancers diagnosed in HICs, such as the United States, or in other regions of Africa (6).

The calculated 3-year survival rate in this study was only 52%. This is consistent with the poor survival rates from breast cancer reported in other sub-Saharan countries, likely due to the initial presentation of locally advanced disease (7, 10). Based on the CONCORD-3 program, which provides metrics to compare the effectiveness of cancer care across 85 countries, from 2010 to 2014, the overall 5-year survival for breast cancer in Ibadan, Nigeria was 97.5% (range 89.9%−100%) (10). Nevertheless, in a recent meta-analysis of breast survival rates in Africa, survival rate in Nigeria (33%) was comparable to that of Ghana (40%) and the rest of sub-Saharan Africa (48%) (2). Additional substantiated drivers of low breast cancer survival in LMICs include: multifactorial delayed individual health-seeking behavior, social stigma, low socio-economic circumstances, multifaceted limits of access to the health system (such as lack of diagnostic and therapeutic infrastructure and capacity of early breast cancer detection), and lack of resource prioritization for treatment of non-communicable diseases (11–13). The time until complete pathologic assessment in this study was up to 9 months, causing delays in critical systemic treatments or forcing the treatment to be given without knowledge of the biological subtype, decreasing the likelihood of therapeutic response. In addition, non-standardized and delayed histological preparation can degrade the surgical specimen's gene and protein expression patterns, falsely underestimating the cancer's ER, PR, and HER2 expression (14). College of American Pathologists (CAP) guidelines recommend a fixation time of <72 h, optimally <24 h, for estrogen and progesterone receptors and <48 h for HER2 receptor evaluation (15).

Many patients who had to pay for adjuvant treatment out-of-pocket failed to complete the prescribed number of cycles of chemotherapy or had a longer than recommended time intervals between treatments. Although radiation was indicated based on the advanced cancer stage, due to the high cost and distance to treatment facilities, radiotherapy was only utilized in 20% of cases. Future studies can be performed to demonstrate that if such treatment modalities were included under the basic medical insurance coverage, it would decrease not only the individual economic impact but also the national economic burden by improving survival in this young demographic, allowing them to care for their families and participate in the work force.

Half of the women in our study were diagnosed at 40 years or younger, two decades younger than the median age at diagnosis in HICs, such as the United States (US) (16). Patients in this rural region of Ghana presented at a younger age, with late-stage disease and a higher rate of the most aggressive cancer biology, which predicted the substantially higher mortality rates observed. In the US, black women are more likely than whites to be diagnosed with breast cancer under the age of 40 and also have twice the incidence of TNBC (6). Black women are also more likely to present with advanced disease when compared with white patients (16–18). It is not well-understood whether these differences in age of onset and tumor biology are caused by specific genetic differences between blacks and whites. However, this study does reinforce the established empirical evidence and support the premise that black women need to be educated about the signs of breast cancer and offered screening for breast cancer beginning at a younger age than Caucasians, Hispanics or Asians.

Despite only a quarter of our patient population having received comprehensive pathologic assessment including immunohistochemistry, the 57% rate of triple-negative (TNBC) disease is consistent with prior peer-reviewed studies where over one-half of patients were diagnosed with TNBC, suggesting a hereditary risk factor for this most aggressive variant of breast cancer (19). The TNBC phenotype has been shown to be more prevalent among people of African descent when compared with Caucasian populations (20). In a comparative analysis by Jiagge et al., (21) among patients younger than 50 years of age, the prevalence of TNBC was highest among Ghanaians (50.8%) and African Americans (34.4%) compared with White Americans and Ethiopians (16% each). Oncologic anthropology studies suggest that the high incidence of hormone receptor-negative breast cancer (and younger age of diagnosis) in West Africa mirrors the age and racial TNBC discrepancy of related black populations in HICs (6). This may be a long-term effect of the transatlantic slave trade when most African slaves originated from West Africa, with many coming directly from ports in Ghana. Future studies of TNBCs may assess the cancers found in both populations of African descent in HICs and Africans in sub-Saharan Africa to look for common genomes that may allow the development of better targeted therapies. Despite elucidating the shared ancestry between West Africans and HIC populations of African descent, it is essential to note that no pathological, genetic, and prognostic differences in TNBC tumors between African descent and Caucasians populations have been identified to date (20). This intrinsic similarity, regardless of race, is important to acknowledge as risk reduction strategies and targeted therapy continue to be improved and shared for the TNBCs that occur both in HICs and in LICs, like Ghana. Our study was not powered to calculate the survival probabilities by tumor phenotype. However, 50% of the patients who were documented to have died also had had immunohistochemistry demonstrating TNBC, suggests an association with the high mortality rate seen in our study. Due to the availability of inexpensive tamoxifen, those patients who had known hormone receptor-positive tumors were prescribed anti-estrogen therapy though we have no data on compliance or duration. For HER2+ cancers, very effective targeted therapies have been developed and are routinely used in HICs with marked improvement in survival of women with HER2 positive cancers. However, due to fiscal constraints, trastuzumab and other HER2 targeted agents are not available to patients in rural Ghana (22).

### Study Limitations and Strengths

With this being the first analysis of breast cancer in a predominantly rural setting, it may be difficult to extrapolate the results to other rural areas of either Ghana or other in sub-Saharan Africa. In addition, 46% of our patients were lost to follow-up and therefore censored. This could have over or underestimated the cumulative survival in this study. Lastly, sub-analysis of survival by biologic subtype could not be done due to a lack of statistical power in the analysis of the results from the limited number of patients who had complete immunohistochemistry analysis available. However, the major strength of our study is that it is the first to demonstrate a low breast cancer survival rate in a rural population in the sub-Saharan African country, Ghana. Another strength is that this data adds to the growing body of literature in identifying high rates of the most aggressive breast cancer subtype, TNBC, in the black population of Ghana.

## Conclusions

Breast cancer survival in sub-Saharan Africa remains poor. Under the Breast Health Global Initiative guidelines, Eastern Regional Hospital's resources fall between that of limited to basic level. The initial patient presentation of advanced breast cancer combined with sub-standard capacity for accurate diagnosis, cancer subtype analysis, adequate therapy and follow-up play a role in the poor outcomes of patients with breast cancer. Future studies should emphasize identification of barriers to care, genetic risk factors, and opportunities for cost-effective and sustainable improvements in LICs.

## Data Availability Statement

The raw data supporting the conclusions of this article will be made available by the authors, without undue reservation.

## Ethics Statement

The studies involving human participants were reviewed and approved by Pennsylvania State College of Medicine Institutional Review Board and Ethical Review Board of Mount Crest University and Eastern Regional Hospital. The patients/participants provided their written informed consent to participate in this study.

## Author Contributions

PS, FA-M, and JO originated the concept and designed experiments. FA-M and XC collected data. PS performed statistical analysis and drafted the manuscript. PS, FA-M, JO, AS, WW, and DD performed critical reviews of the manuscript. All authors approved the final manuscript.

## Conflict of Interest

The authors declare that the research was conducted in the absence of any commercial or financial relationships that could be construed as a potential conflict of interest.

## Publisher's Note

All claims expressed in this article are solely those of the authors and do not necessarily represent those of their affiliated organizations, or those of the publisher, the editors and the reviewers. Any product that may be evaluated in this article, or claim that may be made by its manufacturer, is not guaranteed or endorsed by the publisher.

## Abbreviations

LIC: low-income country
LMIC: low-middle-income countries
HIC: high-income countries
IHC: immunohistochemistry
BSE: breast self-examination
TNBC: triple-negative breast cancer.
